# Sexual Neurasthenia

**Published:** 1884-09

**Authors:** 


### Book Reviews.

---

SEXUAL NEURASTHENIA, With a Chapter on Diet for the Nervous. (Posthumous.) By George M. Beard, A. M., M. D. Edited by A. D. Rockwell, A. M., M. D. E. B. Treat, 757 Broadway, N. Y., 1884.

This work of Dr. Beard on Sexual Neurasthenia is a strange production. It is the most curious medical book to be found. I look upon this departure with its accompaniments like the ringing of bells, the blowing of whistles and the sounding of fog-horns to the mariner who cannot see the vessels which are moving hither and thither around him. In other words, he is befogged upon a dangerous sea, and though using every available means to stem his course through the perils which beset him, he bumps hard upon the ledge of a breaker now and then, or has his bows crushed by a passing vessel that was not discovered in time to avoid the crash. Yet, withal, he makes his way to an open sea under a clear sky, and recounts his marvelous adventures in terms of congratulation for himself and for all concerned. Hurrah for us! is the prominent element that pervades all this erratic disquisition; and the reader, almost against his will, is compelled to admit that, if there are some unwarrantable deductions in his generalizations, yet there are many gleams of light which illumine the obscurities of sexual neurology. The flippant style of the author does not comport well with the enunciation of grave scientific facts and principles, and the sketchy manner in disposing of points involving serious questions must prove a barrier to the acceptance of statements otherwise entitled to the consideration of medical men. There seems to be a tendency at present to popularize medical writings, and unfortunately to adopt the style of light literature, which is sapping the foundation of English composition. There can be no objection to presenting truth in a pleasing manner, and elegance of diction is entirely consistent with the terse style in dealing with scientific subjects, yet the idiom suffers by the coinage of words and distortion of phrases.

The fundamental feature of this production from beginning to end is a far-fetched accommodation of facts to preconceived theory, and overriding all observation and experience, to make the realities of life quadrate with the doctrine of evolution. If the reader will patiently analyze the details given in connection with the report of cases, it will be seen that the data are studiously presented for the corroboration of the prime element in the train of disorders connected with impaired nerve power, and the announcement that "nervousness is really nervelessness," runs through the entire work. The influence of exalted nerve power upon the organization is not recognized as a source of trouble in the class of sexual disorders to which the attention of the author is directed, and he does not lead the reader to suppose that derangement may ensue from this source. As the title limits the sphere of inquiry to sexual disturbance connected with neurasthenia, it might be allowed that no extended notice should be given of such cases as might be associated with hyperesthesia of the nervous system, yet the inference to be drawn from this entirely one sided view of diseased action, excludes over action of the nervous system as an element of sexual derangement.

Without acquiescing in this limitation of derangements of the reproductive system, we note the partial exceptions to the prevailing influence of neurasthenia in some cases of neuralgia, rheumatism and epilepsy, which it is allowed may be accompanied by exalted nerve action.

That the departures from the normal state of the nerves may be more frequent on the side of depression or impaired power than of exaltation or increased action, may be inferred from the general law that all disturbance in the performance of any function whether vital and organic, or simply chemical and mechanical, interferes with its regular and efficient development. Yet, it is a well ascertained physiological fact, that any sort of impediment to the proper action of one part of the physical organization, increases the burden upon others, and that a corresponding augmentation of the forces in such parts is requisite to compensate for the destruction of the equilibrium of the whole animal structure. If, for instance, an artery is ligated in one of the limbs of the body, the circulation must be carried on for the time by the bloodvessels distributed to other parts, so that an increment of force or excitement ensues to provide for the passage of the blood, not simply along accustomed lines, but through new channels opened up by the anastomosing vessels. For the additional work to be done, additional force is requisite, and there is excitement or exaltation of the nervous system to meet this demand. The nervous system is the "*fons et origo*" of all power in the organism, and may become a source of trouble by exceeding or falling short of the normal action.

Apart from the somewhat indefinite relations of this peculiar adynamic phase of the nervous system to sexual disorders, the book is nothing more or less than a very general summary of the author's views upon self abuse, sexual excesses, spermatorrhœa, and their consequences, with suggestions as to medical and surgical treatment, regimen and diet. The observations made in regard to the mooted question of the presence of spermatozoa in the urine of the married and unmarried, leads to the conclusion that "it is a very frequent symptom in all kinds of neurasthenia as well as in many other debilitating diseases."

Great stress is laid upon mental therapeutics and upon muscular exercise, along with a proper discrimination in the use of general sedatives and tonics, but "superior to any, and in many cases to *all* other forms of general tonic treatment in neurasthenic conditions, are the methods known as general faradization and central galvanization."

The war upon vegetable food at the close of the volume is directly at variance with the practical results of Dr. Allison's personal experiments, recently reported, and it must appear somewhat inconsistent with itself when he states that the majority of those who have been suffering for any considerable period, to whom he proposes a diet list based upon his principles, reply, "I am living in that way already." Yet with appropriate modifications to suit different cases, and the elimination of such phrases as "pork has long been driven from our tables," we leave this evolution bill of fare to its chances for a place with the "survival of the fittest."

